# Transduction of Skeletal Muscles with Common Reporter Genes Can Promote Muscle Fiber Degeneration and Inflammation

**DOI:** 10.1371/journal.pone.0051627

**Published:** 2012-12-12

**Authors:** Catherine E. Winbanks, Claudia Beyer, Hongwei Qian, Paul Gregorevic

**Affiliations:** 1 Division of Metabolism and Obesity, Baker IDI Heart and Diabetes Institute, Victoria, Australia; 2 Department of Neurology, The University of Washington School of Medicine, Seattle, Washington, United States of America; 3 Department of Biochemistry and Molecular Biology, Monash University, Melbourne, Australia; 4 Department of Physiology, The University of Melbourne, Melbourne, Australia; University of Minnesota Medical School, United States of America

## Abstract

Recombinant adeno-associated viral vectors (rAAV vectors) are promising tools for delivering transgenes to skeletal muscle, in order to study the mechanisms that control the muscle phenotype, and to ameliorate diseases that perturb muscle homeostasis. Many studies have employed rAAV vectors carrying reporter genes encoding for β-galactosidase (β-gal), human placental alkaline phosphatase (hPLAP), and green fluorescent protein (GFP) as experimental controls when studying the effects of manipulating other genes. However, it is not clear to what extent these reporter genes can influence signaling and gene expression signatures in skeletal muscle, which may confound the interpretation of results obtained in experimentally manipulated muscles. Herein, we report a strong pro-inflammatory effect of expressing reporter genes in skeletal muscle. Specifically, we show that the administration of rAAV6:hPLAP vectors to the hind limb muscles of mice is associated with dose- and time-dependent macrophage recruitment, and skeletal muscle damage. Dose-dependent expression of hPLAP also led to marked activity of established pro-inflammatory IL-6/Stat3, TNFα, IKKβ and JNK signaling in lysates obtained from homogenized muscles. These effects were independent of promoter type, as expression cassettes featuring hPLAP under the control of constitutive CMV and muscle-specific CK6 promoters both drove cellular responses when matched for vector dose. Importantly, the administration of rAAV6:GFP vectors did not induce muscle damage or inflammation except at the highest doses we examined, and administration of a transgene-null vector (rAAV6:MCS) did not cause damage or inflammation at any of the doses tested, demonstrating that GFP-expressing, or transgene-null vectors may be more suitable as experimental controls. The studies highlight the importance of considering the potential effects of reporter genes when designing experiments that examine gene manipulation *in vivo*.

## Introduction

Recombinant adeno-associated viral vectors (rAAV vectors) have been extensively developed as a means of delivering gene expression cassettes *in vivo* to a variety of post-mitotic cell types with the ultimate purpose of ameliorating disease symptoms [1]. The capacity of rAAV vectors to achieve strong and long-lasting transduction of non-dividing cells without significant pathogenicity or genomic integration has also made them valuable tools for manipulating and elucidating gene function in animal models. To this end, rAAV vectors have shown promise as prospective interventions for understanding and treating a variety of conditions affecting the neuromuscular, cardiac, respiratory, hepatic, circulatory and sensory systems [2].

In experiments using rAAV vectors to manipulate gene function, reporter genes such as β-galactosidase [3], [4], [5], human placental alkaline phosphatase (hPLAP) [4], [6], [7], luciferase [8], [9] and green fluorescent protein (GFP) [10], [11] are commonly used as experimental controls. Vectors carrying reporter genes not normally expressed in muscle offer a measure of transduction efficiency, and dose and time dependent effects of transgene expression, while controlling for the influence of administering an equivalent dose of recombinant viral vectors as used in the experimental condition. However, the expression of such non-native genes in skeletal muscle may alter cellular function and therefore complicate the interpretation of effects attributed to delivery of an experimental vector, if used as an experimental control.

Previous studies have observed inflammatory responses in mammalian skeletal muscle following administration of rAAV vectors carrying expression cassettes encoding non-native genes such as bacterial β-galactosidase [3], [12], [13], [14] and coagulation factor IX [15]. However, effects appear to vary by gene, as we, and others have successfully employed rAAV vectors to transduce mammalian skeletal muscle with genes encoding for proteins not normally expressed in the host species [16], [17]. Other groups have reported that over-expression of native proteins can cause toxic effects in skeletal muscle, suggesting that the level of transgene expression may be determine whether cellular breakdown and local inflammation is caused by perturbation of functions within the target cell, as an alternative to activation of immunogenic responses [18]. Given that recombinant AAV vectors are capable of achieving highly effective delivery of gene expression cassettes, and that reporter gene-based vectors are routinely used as “non-functional” controls when experimentally manipulating muscle, it is important to ascertain whether commonly used reporter transgenes can elicit effects of their own when expressed in skeletal muscle.

To answer this question, we examined murine hind limb muscles following administration of pseudotyope-6 rAAV vectors carrying expression cassettes encoding for hPLAP and GFP reporter genes. We report herein that local administration of rAAV6:hPLAP vector to skeletal muscle causes dose- and time-dependent pro-inflammatory macrophage recruitment as well as significant skeletal muscle damage. These effects were independent of promoter type, as vectors carrying expression cassettes featuring constitutive CMV and muscle-specific CK6 promoters drove a similar cellular response when matched for total genome numbers. The observed deleterious effects were related to the level of expression of the specific transgene, as we observed that an increased dose of rAAV6 vectors carrying a GFP expression cassette was required to produce a similar inflammatory/degenerative response. Moreover, at all vector doses tested, rAAV vectors carrying a gene-less expression cassette were well tolerated. We conclude that studies employing reporter gene constructs as experimental controls in the study of mammalian skeletal muscle should consider the impact of the reporter gene upon target cell function before interpreting the effects of an experimental intervention in comparison to a muscle transduced with a reporter gene.

**Figure 1:**
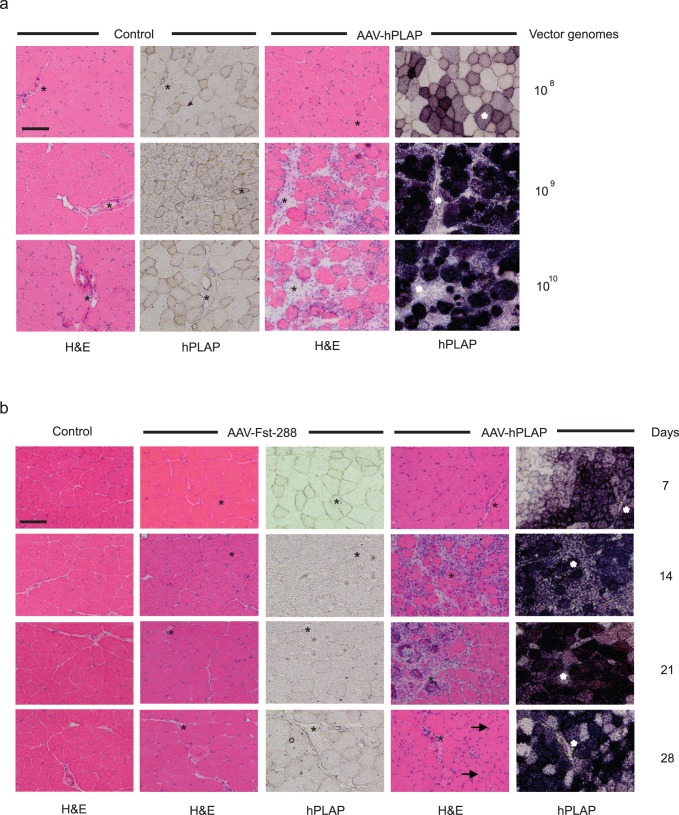
Intramuscular administration of rAAV6:CMV-hPLAP vectors induces skeletal muscle inflammation and damage in a dose and time-dependent manner. (a) The TA muscles of mice were injected with either 1×10^8^, 1×10^9^ or 1×10^10^ genomes of the control vector, or rAA6:CMV-hPLAP and examined 14 days afterwards. TA muscles were dissected and stained with Hematoxylin & Eosin for general morphology, or with NBT/BCIP to determine the expression of human placental alkaline phosphatase (purple). Asterisks identify common features on the serial sections used for morphology and hPLAP activity. (b) A time-course analysis of muscles examined 7, 14, 21 and 28 days after injection of rAAV6 vectors indicates peak times of induction of inflammation in response to rAAV:CMV-hPLAP, as compared to a gene-less vector (rAAV6:CMV-MCS) or rAAV6:Follistatin288.

## Methods

### Ethics Statement

All experiments using animals were conducted in accordance with the relevant codes of practice for the care and use of animals for scientific purposes (National Institutes of Health, 1985, and the National Health & Medical Council of Australia, 2004). All experimental protocols were approved by the Alfred Medical Research and Education Precinct Animal Ethics Committee (AMREP AEC). All surgery was performed under inhalation of isoflurane in medical oxygen, and stringent protocols were followed to minimize pain and discomfort.

**Figure 2:**
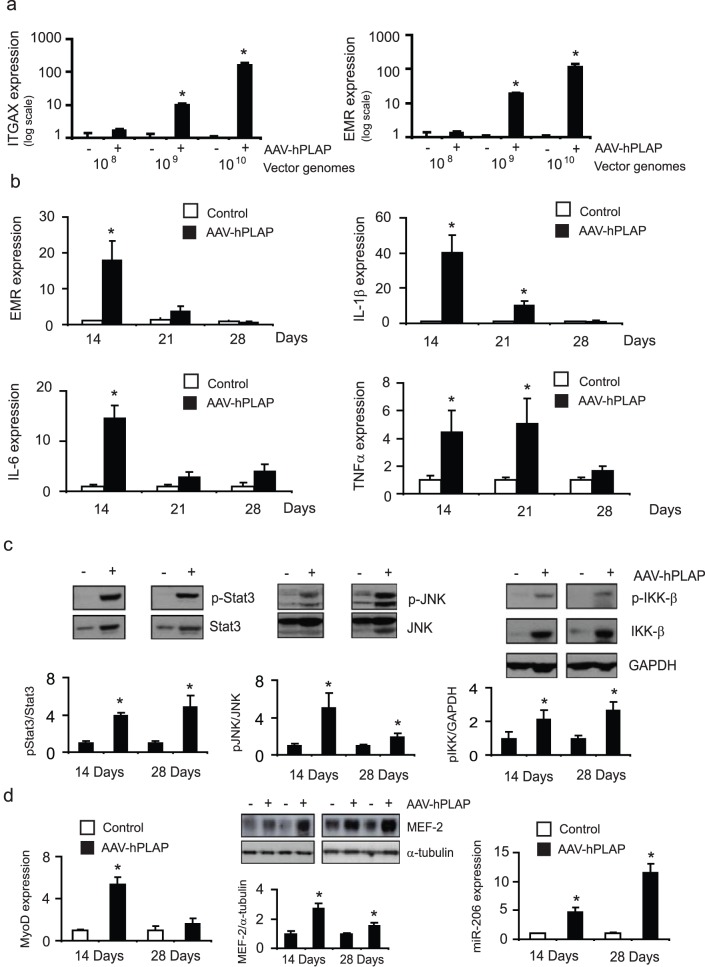
Administration of rAAV6:CMV-hPLAP results in increased expression of pro-inflammatory macrophages markers, and pro-inflammatory signaling pathway activation. (a) TA muscles were injected with the indicated doses of rAAV6:CMV-hPLAP or rAAV6:CMV-MCS control. At 14 days post-injection, RNA was extracted from muscle tissue and EMR1 and ITGAX expression was analyzed. *, p<0.05 vs. control. (b) EMR expression, as well as other markers of inflammation, IL-1β, IL-6 and TNFα were analyzed over 14, 21 and 28 days after administration of 1×10^9^ genomes of rAAV6:CMV-hPLAP. *, p<0.05 vs. control. (c) The upregulation of IL-6 expression also correlates with increased phosphorylation of Stat3. JNK and IKK-β phosphorylation were also assessed by Western blot *, p<0.05 vs. control. (d) MyoD and miR-206 gene expression, as well as MEF-2 protein levels, were analyzed from tissue harvested 14 or 28 days after vector administration. *, p<0.05 vs. control.

### Chemicals and Antibodies

All antibodies were obtained from Cell Signaling Technology except GAPDH and MEF-2 (Santa Cruz). The expression of hPLAP was examined on cryosections using a NBT/BCIP substrate solution (Sigma FAST, Sigma). Other chemical reagents were obtained from Sigma unless otherwise stated.

**Figure 3:**
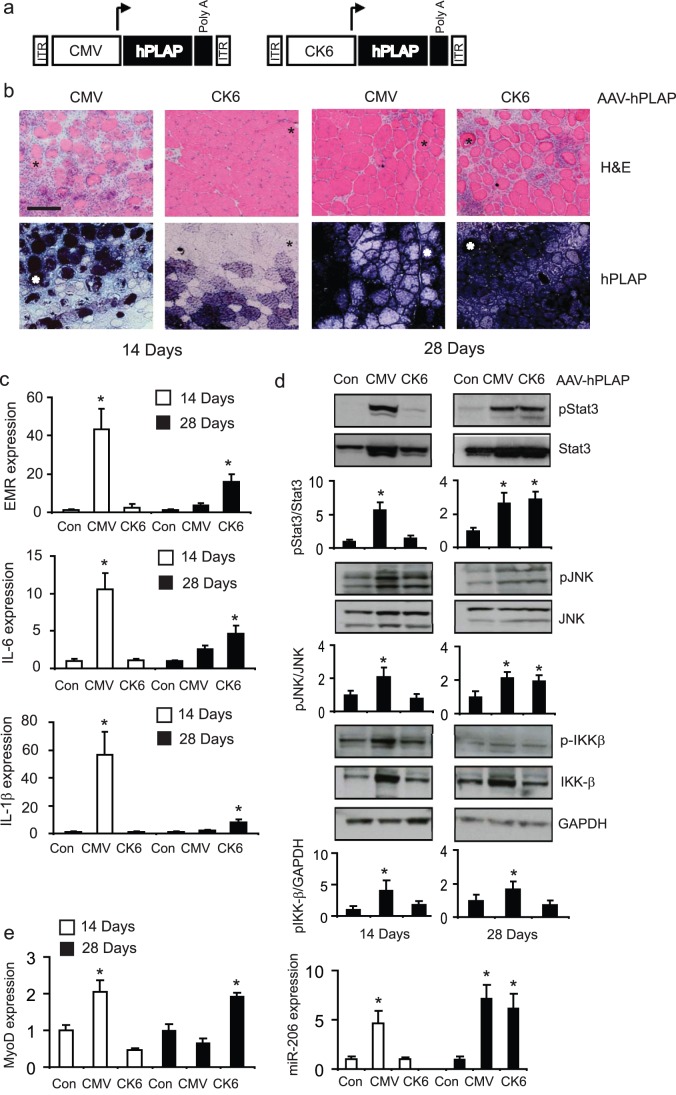
Substitution of rAAV6:CMV-hPLAP with a muscle-specific CK6 promoter does not ameliorate the effects of vector-mediated hPLAP expression on muscle damage and inflammation (a) Designs of expression cassettes packaged into rAAV6:CMV-hPLAP and rAAV6:CK6-hPLAP vectors (b) Vectors were injected into the TA muscles of mice, and their effects examined 14 or 28 days afterwards, using the same methods as in **Figure 1**. Damage and cellular infiltration was not evident in muscles examined 14 days after injection with rAAV6:CK6-hPLAP, but was notable by 28 days post-injection. (c) EMR, IL-6 and IL-1β expression were assessed at 14 and 28 days after administration of rAAV6:CMV-MCS, rAAV6:CMV-hPLAP and rAAV6:CK6-hPLAP vectors. *, p<0.05 vs. control (d) Protein was extracted from muscles and phosphorylation levels of inflammatory mediators Stat3, JNK and IKK-β were determined by Western blot analysis. *, p<0.05 vs. control (e) MyoD and miR-206 expression was examined in muscles collected 14 or 28 days after administration of rAAV6:CMV-MCS, rAAV6:CMV-hPLAP and rAAV6:CK6-hPLAP vectors. *, p<0.05 vs. control.

### Cloning and Production of Recombinant Adeno-associated Viral Vectors

cDNA constructs encoding human PLAP, humanized Renilla GFP or human follistatin-288 were cloned into an AAV expression plasmid consisting of a CMV or CK6 promoter/enhancer [19], [20] and SV40 poly-A region flanked by AAV2 terminal repeats [21], using standard cloning techniques. Transfection of these plasmids with the pDGM6 packaging plasmid into HEK293 cells generated type-6 pseudotyped viral vectors that were harvested and purified as described previously [21]. Briefly, HEK293 cells suspended in Dulbecco’s Modified Eagle’s Medium (DMEM, Life Technologies) supplemented with 10% fetal bovine serum (Hyclone) and 1% penicillin/streptomycin (Life Technologies) were plated at a density of 3.2−3.8×10^6^ cells on sterile 10 cm culture dishes (Corning), 8−16 h prior to transfection with 10 µg of an AAV:expression cassette plasmid and 20 µg of the packaging/helper plasmid pDGM6, by means of the calcium phosphate precipitate method to generate pseudotype 6 vectors [21]. At 12 hours after transfection, the cells commenced incubation in serum-free media, and at 72 hours after transfection, the media and cells were collected and homogenized through a microfluidizer (Microfluidics Inc) prior to 0.22 µm clarification (Millipore). The vectors were recovered from the clarified lysate by affinity chromatography over a heparin affinity column (HiTrap, GE Life Sciences) and ultracentrifuged overnight prior to re-suspension in sterile physiological Ringer’s solution. The purified vector preparations were quantified with a customized sequence-specific quantitative PCR-based reaction (Applied Biosystems), consisting of a forward primer (ttttcactgcattctagttgtggtt), reverse primer (catgctctagtcgaggtcgagat) and probe (6FAM-actcatcaatgtatcttatcatg-MGBNFQ).

**Figure 4:**
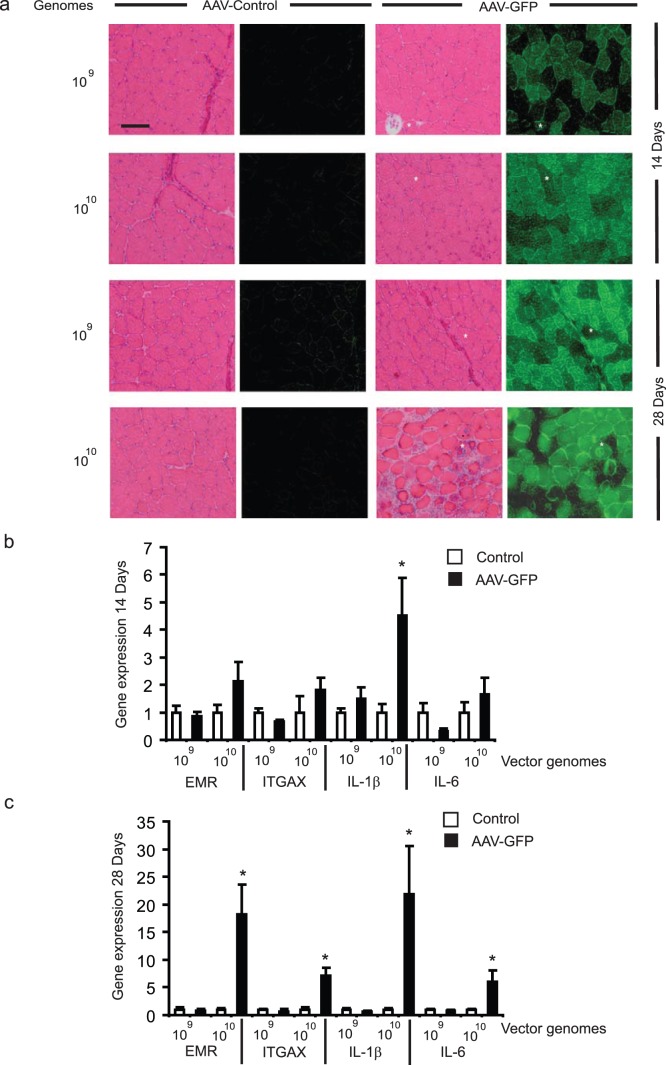
rAAV6 vector-mediated expression of GFP exerts a reduced inflammatory effect in skeletal muscle compared with expression of hPLAP. (a) rAAV6:CMV-GFP or “gene less” rAAV6:CMV-MCS vectors were injected into the TA muscles of mice at 1×10^9^ or 1×10^10^ genomes. Muscles examined 14 and 28 days after injection of 1×10^9^ rAAV6:CMV-GFP vector genomes demonstrated strong transgene expression without evidence of cellular infiltration, or muscle breakdown. However inflammation was evident in muscles examined 28 days after receiving 1×10^10^ vg of rAAV6:CMV-GFP. (b-c) Expression of EMR, ITGAX, IL-1β and IL-6 was not different in muscles examined 14 or 28 days after receiving 1×10^9^ vg of rAAV6:CMV-GFP (compared with muscles receiving rAAV6:CMV-MCS) but was elevated in muscles examined 14 or 28 days after receiving 1×10^10^ vg of rAAV6:CMV-GFP. *, p<0.05 vs. control.

### Experimental Animals and Surgical Procedure

For local vector delivery, ∼8 wk old male C57BL/6 mice were deeply anesthetized, and 1×10^8^, 1×10^9^ or 1×10^10^ genomes of a given vector in 30 µl of Hank’s buffered saline solution (HBSS) were injected directly into the tibialis anterior (TA) muscle occupying the anterior compartment of the lower hind limb, via a reusable syringe equipped with 32 g needle (Hamilton). Control-injections of the contralateral limb muscle used a vector lacking a functional gene (referred to as rAAV6:MCS). For tissue harvest, mice were humanely killed via a cervical dislocation, and the TA muscles rapidly excised, blotted dry and weighed, before subsequent processing.

### Histochemical Staining

Freshly harvested muscles were placed in optimal cutting temperature cryoprotectant (Tissue-Tek OCT, Sakura) and frozen in liquid nitrogen-cooled isopentane. The frozen samples were subsequently cryosectioned at 10 µm thickness and stained with hematoxylin and eosin to examine morphology as described previously [22]. Sections were fixed in methanol, rinsed in distilled water, immersed in hematoxylin solution (Amber Scientific, Australia) for 3 minutes, dip-rinsed in distilled water and tap water, incubated in Scott’s tap water (Amber Scientific, Australia) for 1 minutes, followed by running tap water for 2 minutes, then immersed in Eosin solution (Amber Scientific, Australia) for 2 minutes, and subsequently transferred through increasing strengths of ethanol before immersion in xylene, and cover-slipping with DEPEX (BDH) mountant. Histochemical staining for hPLAP activity was conducted as described previously [6]. Briefly, sections were fixed with 4% paraformaldehyde, washed three times in cold phosphate buffered saline (PBS), placed in 65°C PBS for 90 minutes and rinsed in room temperature PBS. Excess liquid was removed from the sections, and NBT/BCIP substrate solution (Sigma) was applied to each section for 30 minutes at room temperature in the dark. Slides were rinsed three times in PBS and cover-slipped with Permount™ mounting media (Fisher Scientific).

### Real Time QPCR

Total RNA was collected from snap frozen tissue following homogenization in ice-cold Trizol (Invitrogen). 500–1000 ng of RNA was reverse transcribed using the High Capacity RNA-to-cDNA kit (Applied Biosystems). cDNA was subsequently analyzed by quantitative RT-PCR using target specific probe and primer sets for EMR1, ITGAX, TNFα, IL-1β MyoD and IL-6 (Taqman™, Applied Biosystems) alongside a probe and primers for 18S used to standardize for cDNA concentrations. For miR-206 analysis, Assay on Demand™ reagents (Applied Biosystems) were used according to the manufacturer’s instructions. Data were analyzed using the ΔΔCT method of analysis and are presented as fold change to a control value of 1.

### Western Blotting

TA muscles were homogenized in RIPA-based lysis buffer (Merck Millipore) with EDTA-free protease and phosphatase inhibitor cocktails (Complete™ Tablets, Roche). Lysis was followed by centrifugation at 13000×g for 10 min at 4°C and samples were denatured for 5 min at 95°C. Protein concentration was determined using a micro protein assay kit (Pierce, Thermo Scientific). Protein fractions were subsequently separated by SDS-PAGE using pre-cast 4–12% Bis-Tris gels (Invitrogen), blotted onto nitrocellulose membranes (Biorad) and incubated with the appropriate antibody overnight. All primary antibodies were made up at 1∶1000 dilutions in PBS-tween containing 5% BSA, except GAPDH (used at 1∶5000). Membranes were washed and incubated in the appropriate secondary (used at 1∶5000) for 1 hour at room temperature. Membranes were then developed as described previously [23]. Quantification of labeled Western blots was performed using ImageJ pixel analysis (NIH Image software) [24], and data are normalized to a control value of 1. Densitometric analyses of Western blots are presented as band density normalized to the loading control, and are representative of at least three independent experiments.

### Statistical Analysis

The Student T-test was used to assess differences in one variable between two groups. One-Way ANOVA was used to assess differences in multiple groups, whilst the Student-Newman-Keuls post-hoc test was used for comparisons between groups. Data are presented as the mean±S.E.M.

## Results

### rAAV6:CMV-hPLAP Administration Induces Inflammation in Murine Skeletal Muscles in a Dose- and Time-dependent Manner

hPLAP has been used as a reporter gene in previous studies to determine transduction efficiency, and also as an experimental control when investigating the effects of manipulating genes of interest using vector-mediated gene delivery. When transduced into the TA muscles of mice, expression of protein from this transgene induces dose-dependent increases in inflammation and muscle damage which correlate with increases in hPLAP activity 14 days after rAAV6:hPLAP administration (Fig. 1a). Whilst no inflammatory response was observed from the lowest viral genome dose employed here, the local administration of 1×10^9^ vector genomes (or higher doses) was associated with marked evidence of cellular infiltration and tissue damage. This effect was also time-dependent, as inflammation and tissue damage that was evident 14 days after the administration of 1×10^9^ vector genomes was not observed in muscles examined 7 days after vector delivery (Fig. 1b). In muscles expressing high levels of hPLAP when examined 28 days after rAAV6:hPLAP administration, numerous centrally nucleated muscle fibers were observed, suggesting a regenerative response to the prior degeneration of transduced muscle fibers (see arrows). The cellular infiltration and muscle degeneration observed was determined to be a product of hPLAP expression, as expression of another transgene – human follistatin-288 [17], following administration of an equivalent vector dose, did not induce an inflammatory response and disruption of tissue architecture.

### rAAV Vector-mediated Expression of hPLAP Promotes Recruitment of Macrophages and T-cells, and the Activation of Inflammatory Signaling Cascades

To confirm that expression of hPLAP in murine TA skeletal muscle induces a response that is associated with macrophage recruitment, we measured the expression of pro-inflammatory macrophage markers in response to increasing doses of vector administration. Whilst the administration of 1×10^8^ vector genomes did not affect EMR1 or ITGAX expression, administration of 1×10^9^ or more vector genomes led to marked increases in EMR and ITGAX expression in lysates obtained from muscle samples examined after 14 days (Fig. 2a). Of the time points studied, EMR expression peaked at 14 days, and subsided thereafter until 28 days where there was no significant difference when compared to TA muscles injected with rAVA6:CMV-MCS (Fig. 2b).

We next examined other makers of inflammation including the cytokines IL-1β, IL-6 and TNF-α and found that like EMR, their expression was maximal 14 days after rAAV6:CMV-hPLAP administration, and thereafter subsided by 28 days (Fig. 2b). To further confirm activation of pro-inflammatory pathways, we examined Stat3, JNK and IKK-β phosphorylation. Lysates of muscles injected with rAAV6:CMV-hPLAP exhibited increased phosphorylation of Stat3, JNK and IKK-β (Fig. 2c). The upregulation of myogenic regulatory factors is required to facilitate differentiation of newly forming myofibers during muscle regeneration, and their upregulation is therefore a marker of muscle remodeling and repair. Accordingly, we also confirmed that the inflammatory response induced by expression of hPLAP in muscle coincided with regeneration of skeletal muscle fibers as demonstrated by increased levels of MyoD at the gene level, and increased MEF-2 at the protein level. These changes also coincided with the induction of microRNA-206. This signaling circuitry has previously been elegantly demonstrated to regulate cellular differentiation [25], [26] (Fig. 2d).

### Expression of hPLAP under the Control of a Muscle-specific Promoter is also Associated with Degeneration of Murine Musculature and Inflammation

Given the ability of the CMV promoter to potently express transgenes in different cell types, it is unclear from the studies reported here as to whether CMV driven rAAV6:hPLAP is directly transducing, and activating resident inflammatory cells in skeletal muscle. To test this hypothesis, we administered 10^9^ genomes of rAAV vectors carrying the hPLAP expression cassette after substituting the CMV promoter with a muscle-specific CK6 promoter, which does not express in tissues other than skeletal muscle [20] (Fig. 3a), and compared the effects of this vector to those observed following administration of rAAV6:CMV-hPLAP (Fig. 3b). Whilst the deleterious effects of rAAV6:CMV-hPLAP upon TA muscle morphology were recapitulated 14 days after vector administration, the injection of rAAV6:CK6-hPLAP did not appear to affect TA skeletal muscle architecture at the same time point. However, by 28 days, inflammation and tissue destruction was evident in TA muscles that had been injected with rAAV6:CK6-hPLAP (Fig. 3b).

When we examined macrophage and inflammatory marker gene expression, we found that injection of rAAV6:CMV-hPLAP vectors had marked effects on the induction of EMR, IL-6 and IL-1β expression at 14 days, whilst injection of rAAV6:CK6-hPLAP did not. However, by 28 days post treatment, when the pro-inflammatory signature had diminished in muscles administered rAAV6:CMV-hPLAP vectors, a definite, albeit reduced increase in these markers was observed in muscles administered rAAV6:CK6-hPLAP vectors. The phosphorylation of inflammatory mediators IKKβ, JNK and Stat3 was also increased in muscles examined 28 days, but not 14 days, after administration of rAAV6:CK6-hPLAP vectors (Fig. 3d). We also confirmed that the cellular disruption observed after administration of rAAV6:CK6-hPLAP also coincided with increased expression of the regenerative markers MyoD and micro-RNA-206 (Fig. 3e). Changes in MyoD and miR-206 expression were comparable between muscles treated with rAAV6:CK6-hPLAP and rAAV6-CMV:hPLAP. These data demonstrate that although expression of hPLAP under the control of the CK6 promoter/enhancer is restricted to skeletal muscle, the level of transgene expression afforded in muscle can also result in inflammation and damage to muscle fibers.

### rAAV6 Vector-mediated Expression of GFP Exerts a Reduced Inflammatory Effect in Skeletal Muscle Compared with Expression of hPLAP

As a means to identify a more suitable reporter transgene, we sought to examine the effect of expressing humanized Renilla GFP in muscles, by administering 1×10^9^ or 1×10^10^ rAAV6:CMV-GFP vectors to the TA muscles of mice. When rAAV6:CMV-GFP was administered at a dose of 1×10^9^ vector genomes, we found that significant GFP expression was achieved in transduced hind limb muscles, but that the architecture of murine muscles was preserved for at least 28 days (Fig. 4a). Only when we increased the dose of rAAV6:CMV-GFP administered by ten fold (1×10^10^ genomes) was significant muscle damage accompanied by cellular infiltration observed. In subsequent assessments of markers for macrophage infiltration and inflammation, we found no significant marker induction when muscles received 1×10^9^ rAAV6:CMV-GFP vector genomes (in contrast to the effects noted with an equivalent dose of rAAV6:CMV-hPLAP). However, when rAAV6:CMV-GFP was administered at a dose of 1×10^10^ vector genomes, significant increases in macrophage and inflammatory markers were detected after 28 days (Fig. 4b). These data indicate that the use of GFP may be a better alternative to hPLAP as a reporter gene for expression in skeletal muscle, but that vector dose, and the magnitude of ensuing transgene expression must be taken into account during experimental design.

## Discussion

When using recombinant AAV vectors to manipulate gene expression in skeletal musculature, parallel cohorts are often treated with vectors carrying reporter genes as experimental controls. While reporter genes may be regarded as “non-functional” compared with experimental constructs of interest, it is important to consider the effects of the reporter gene when contemplating experimental design, and the relative interpretation of experimental interventions. In this study, we have shown that genes commonly delivered in reporter constructs can promote dose-dependent inflammation and breakdown of murine skeletal musculature. The findings demonstrate that the choice of reporter gene and degree of expression are important considerations when designing studies to examine the impact of a vector-based intervention upon cellular processes implicated in muscle adaptation, and the morphological attributes of experimentally manipulated muscles.

Intramuscular inflammation and degeneration of transduced musculature may be caused by priming the immune system to eliminate an introduced antigen, such as the capsid proteins comprising a viral vector particle [27]. Prior exposure of humans and other mammals to wildtype adeno-associated viruses or rAAV vectors can sensitize a host’s immune system to reaction against subsequently administered vectors [28], [29]. However we and others have extensively demonstrated that recipients not previously exposed typically tolerate intramuscular administration of rAAV vectors without evidence of cellular damage [17]. Recombinant AAV vectors typically exert very little evidence of adverse effects upon target cells, as they lack the coding regions of their wildtype genome, are derived from wildtype viruses that are not associated with specific human pathologies, and typically do not promote modification of the host cell’s genome. Our data are consistent with previous findings, as we were able to directly administer rAAV vectors lacking a functional gene (rAAV6:CMV-MCS) to murine musculature without causing ensuing cellular damage and inflammation.

The transduction of skeletal muscles with constructs expressing non-native proteins can also promote an immune reaction and associated tissue damage, as this has been demonstrated following intramuscular administration of rAAV vectors [30], [31]. However, this response appears to vary depending on the gene being expressed, as many other studies (including work of our own) have employed rAAV vectors to successfully transduce mammalian musculature with constructs encoding for non-native genes without observing ensuing tissue damage and inflammation [4], [16], [32]. In our studies reported here, we have shown similarly well-tolerated expression of non-native transgenes, by using rAAV vectors to express human follistatin-288 in murine skeletal muscles. We have also achieved robust expression of Renilla-derived green fluorescent protein in murine skeletal muscles without evidence of cellular degeneration and inflammation, depending on the vector dose used.

Our findings of a positive correlation between rAAV6:hPLAP vector dose and the incidence of inflammation and cellular damage in murine muscles (and a similar correlation albeit requiring higher doses for rAAV6:GFP) suggest that specific gene products may perturb cellular function if expressed at sufficiently high levels. In support of this idea, it has been reported that dose-dependent toxic effects can be observed even after expressing muscle-specific transgenes in skeletal muscle via vector based approaches [18]. Some studies have used tissue-specific promoter/enhancer elements to reduce toxicity in transduced musculature and minimize the potential for unintentional transgene expression from antigen producing cells [19], [33], [34], whereas others have reported that the use of muscle-specific promoters does not prevent a deleterious reaction [3], [35]. The inflammatory response we observed in muscles transduced with hPLAP expression cassettes was less-pronounced at early time-points when the CMV promoter was substituted with a muscle-specific, creatine kinase-derived promoter (CK6) [19]. The reduced inflammation induced by hPLAP when driven by the muscle specific promoter correlated with reduced expression levels of hPLAP within TA muscles at this time point. However, significant damage was still observed in muscles treated with rAAV6:CK6-hPLAP at later time points, concomitant with progressive increases in hPLAP expression. Our findings are consistent with previous research in which the inflammatory response to transduction of mammalian musculature was not eliminated but delayed by substituting in a muscle-specific promoter instead of a CMV promoter [3], [35]. The CK6 promoter is considerably less potent in its ability to drive reporter gene expression in skeletal muscle than the CMV promoter [20]. Therefore, in our studies, accumulation of hPLAP to toxic levels would occur more slowly in muscles transduced with CK6-hPLAP constructs, compared with in muscles receiving CMV-hPLAP constructs.

To determine whether an alternative choice of reporter gene might achieve more practical transduction of mammalian musculature without inflammation, we administered rAAV6:CMV-GFP vectors to the muscles of mice. In contrast to results obtained following administration of rAAV6:CMV-hPLAP, we observed that transduction of muscles with an equivalent 1×10^9^ vg dose of rAAV6:CMV-GFP elicited widespread transgene expression without evidence of cellular degeneration or inflammatory response. However increasing the dose of rAAV6:CMV-GFP injected to 1×10^10^ vg subsequently resulted in muscle damage, macrophage recruitment and inflammatory signaling pathway activation. Our data indicate that GFP should be preferred over hPLAP as a reporter gene to express in murine skeletal muscle, but consideration should still be given to the dose of vector employed and the ensuing level of transgene expression caused. In some instances where administration of higher vector doses is warranted, we suggest that it is preferable to employ a gene-deleted vector as an experimental control, as this configuration does not appear to cause the cellular degeneration and inflammation observed following transduction of limb muscles with higher doses of vectors carrying the aforementioned reporter genes.

In summary, our studies highlight the potential deleterious effects of commonly used reporter genes when expressed in mammalian skeletal muscle. Both hPLAP and GFP have the ability to induce robust macrophage recruitment and inflammatory pathway activation in murine muscles, and the effects appear to be related to the level of transgene expression, rather than the vector particle load. Importantly, the potential to cause degeneration and inflammation of transduced muscles also appears to vary between reporter genes. Therefore, it is conceivable that other reporter genes (for instance alkaline phosphatase variants [36], [37], other fluorescent proteins, and luciferase constructs) may have the capacity to cause similar deleterious effects in skeletal muscles if expressed at sufficiently high levels. These findings provide important insight into the potential adverse effects of expressing commonly used reporter genes in mammalian skeletal muscle, and highlight the importance of defining their potential impact upon transduced tissues before being used as experimental controls for *in vivo* studies.
